# Detection of Epileptogenic Cortical Malformations with Surface-Based MRI Morphometry

**DOI:** 10.1371/journal.pone.0016430

**Published:** 2011-02-04

**Authors:** Thomas Thesen, Brian T. Quinn, Chad Carlson, Orrin Devinsky, Jonathan DuBois, Carrie R. McDonald, Jacqueline French, Richard Leventer, Olga Felsovalyi, Xiuyuan Wang, Eric Halgren, Ruben Kuzniecky

**Affiliations:** 1 Comprehensive Epilepsy Center, Department of Neurology, New York University, New York, New York, United States of America; 2 Multimodal Imaging Laboratory, University of California San Diego, San Diego, California, United States of America; 3 Center for Neural Science, New York University, New York, New York, United States of America; 4 Royal Children's Hospital, Murdoch Childrens Research Institute, University of Melbourne, Melbourne, Australia; Brigham and Women's Hospital, Harvard Medical School, United States of America

## Abstract

Magnetic resonance imaging has revolutionized the detection of structural abnormalities in patients with epilepsy. However, many focal abnormalities remain undetected in routine visual inspection. Here we use an automated, surface-based method for quantifying morphometric features related to epileptogenic cortical malformations to detect abnormal cortical thickness and blurred gray-white matter boundaries. Using MRI morphometry at 3T with surface-based spherical averaging techniques that precisely align anatomical structures between individual brains, we compared single patients with known lesions to a large normal control group to detect clusters of abnormal cortical thickness, gray-white matter contrast, local gyrification, sulcal depth, jacobian distance and curvature. To assess the effects of threshold and smoothing on detection sensitivity and specificity, we systematically varied these parameters with different thresholds and smoothing levels. To test the effectiveness of the technique to detect lesions of epileptogenic character, we compared the detected structural abnormalities to expert-tracings, intracranial EEG, pathology and surgical outcome in a homogeneous patient sample. With optimal parameters and by combining thickness and GWC, the surface-based detection method identified 92% of cortical lesions (sensitivity) with few false positives (96% specificity), successfully discriminating patients from controls 94% of the time. The detected structural abnormalities were related to the seizure onset zones, abnormal histology and positive outcome in all surgical patients. However, the method failed to adequately describe lesion extent in most cases. Automated surface-based MRI morphometry, if used with optimized parameters, may be a valuable additional clinical tool to improve the detection of subtle or previously occult malformations and therefore could improve identification of patients with intractable focal epilepsy who may benefit from surgery.

## Introduction

Cortical malformations (CM), particularly focal cortical dysplasia (FCD), are increasingly recognized as the most common etiology in pediatric epilepsy and the second most common etiology in adults with medically intractable seizures [1]. Recent advances in MR imaging have allowed for improved visual detection and diagnosis of FCD and other CM in patients with epilepsy [2]. Common features of FCD include abnormal gyral and sulcal pattern, thickening of the cortical gray matter, blurring of the gray-white matter junction, abnormal neural and glia-derived cell types and high signal on T2 or FLAIR sequences [3], [4]. Despite high-resolution MRI, post-operative histological studies demonstrate that up to 50–80% of FCD escape routine visual inspection [5]. Increased detection of epileptogenic focal CM would provide a valuable surgical target to reduce or eliminate seizures [6], [7]. Further, the most reliable predictor of seizure freedom after surgery for MC is the extent to which the lesion is resected. Incomplete resection decreases the chance of seizure freedom from 77% to 20% [1]. Accurate and sensitive pre-surgical identification of CMs allows for more targeted placement of intracranial EEG electrodes, potentially more complete resection of CMs, and improved surgical outcome. Therefore, methodological advancements that increase sensitivity and accuracy in delineating the presence and extent of FCD and other types of focal CM would be a very valuable addition to current preoperative evaluations. Automated, easy-to-use methods to identify and diagnose neocortical lesions would increase referrals to epilepsy centers for surgical consideration. More than three-quarters of neurologists refer patients with an MRI-identified lesion, but less than half refer non-lesional patients with a presumed extra-temporal focus [8].

Automated MRI brain image analysis techniques, such as voxel-based morphometry (VBM) [9], offer advantages over subjective techniques and have been succesfully employed to detect subtle anatomical anomalies in a single neurological patient compared to healthy controls [10], [11], [12], [13]. VBM is primarily used to detect differences in gray-matter concentration/density, however, recent advancements in volume-based morphometry allow for cortical thickness and gray-white matter gradient estimation. As such, measures that directly quantify anatomical patterns that correlate with histological abnormalities in disorders such as FCD (i.e. cortical thickness, gray-white matter contrast) may show improved detection sensitivity compared to gray matter density [14]. However, because of voxel-wise analysis and volume-based averaging procedures used in VBM, the detection of small, spatially restricted lesions in folded cortex may be limited. In contrast, surface-based coregistration methods align specific cortical sulci and gyri across brains, thus allowing a more precise matching and comparison of anatomical structures across subjects [15].

Cortical thickness measures the distance between white and gray matter surface at multiple points in the brain [16], [17] and is sensitive to changes occurring in health (e.g. aging) and disease (e.g. depression, schizophrenia, Alzheimer's disease, Huntington's disease [18], [19], [20].

Applied to epilepsy, group-wise comparisons of cortical thickness have shown increased region-specific thinning in mesial temporal lobe epilepsy patients compared to normal controls [21]. The present proof of concept study seeks to establish the feasibility of surface-based MRI morphometry to detect, in an unbiased, automated fashion, known areas of structural abnormality in individual patients with a range of epileptogenic cortical malformations. To our knowledge, only one previous study [14] has used a surface-based approach with cortical thickness and contrast measures to detect cortical abnormalities. Here, we extend this method to include additional measures of cortical morphology that are specific to surface-based approaches, such as local gyrification, sulcal depth, jacobian distance and curvature. A further aim is to determine the effects of imaging parameters, i.e. smoothing and statistical threshold, on detection sensitivity and specificity. To verify the surface-based quantitative approach and relate findings to focal epilepsy, this study evaluates results against expert lesion-tracing, location-specific intracranial EEG, histo-pathology and surgical outcome.

## Results

**Figure 1:**
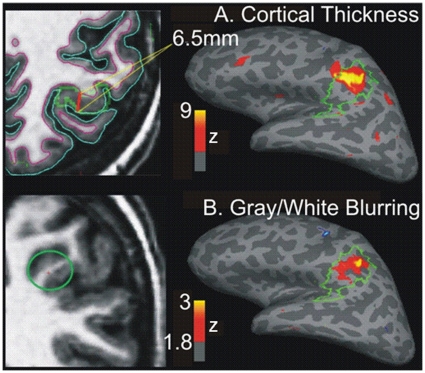
Cortical dysplasia marked by cortical thickening and gray-white boundary blurring in Patient 3. **A.** Significantly increased thickening of the cortical ribbon (in red/yellow) detected by the automated quantitative approach falls within the expert-delineated area of focal cortical dysplasia (green tracing on the inflated pial surface). The area with highest z-score shows cortical thickness measures of up to 6.5 mm between white matter and pial surfaces. **B.** Calculating the T1-signal contrast at .5 mm above vs below the gray/white interface shows an area of significant blurring within the lesion. The green circle on the left shows the area of maximum blurring on a coronal volumetric MRI slice.

**Figure 2:**
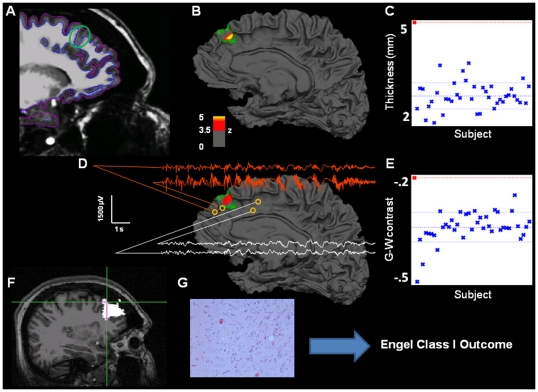
Multimodal results for Patient 4. **A.** Sagittal T1 with pial and white matter surface tracings used for calculating cortical thickness and G–W contrast. Green circle marks area of visually identified focal cortical dysplasia. **B.** Patient's reconstructed white matter surface with area of significant cortical thickening marked in red/yellow (z>3.5, cluster-corrected) within the expert-delineated lesion margin (green tracing). **C.** Mean cortical thickness of the true positive cluster for patient (red cross, top left) and all normal control subjects in blue. **D.** Same as B, but for gray-white matter contrast. The significant cluster shows an area of significant blurring of the gray-white matter junction. EEG traces from intracranial electrodes near the dysplastic area showing focal interictal and ictal discharges (orange), compared to slightly more distant electrode locations (white), showing a co-localization of detected lesion and pathological electrophysiology. **E.** Mean gray-white matter contrast for the single patient and all control subjects. More positive values denote increased gray-white boundary blurring. **F.** Resected area after surgery (in white). Crosshair marks area of maximum thickness increase within the detected lesion. This patient is seizure free one year after surgery.

Quantitative morphometry findings are demonstrated in Figures 1 and 2 from two representative patients. Figure 1 shows the cortical thickness and gray-white matter blurring findings, in relation to the identified malformation, in Patient 3. This patient has not had invasive electroencephalography or resective surgery. Patient 4 was assumed non-lesional before her referral to our Epilepsy Center. The pre-surgical evaluation identified a small area suspected of FCD in the right frontal lobe and scalp EEG demonstrated poorly lateralized ictal onsets. Figure 2 shows the patient's brain surface rendering demonstrating with surface-based morphometry thickened gray matter and poor gray white differentiation at its depth, consistent with FCD. Intracranial electroencephalography localized the ictal onset zone to the right frontal lobe, corresponding to the region identified with quantitative measures. Following resection, the tissue analyses confirmed the diagnosis of cortical dysplasia. This patient has remained seizure free (Engel Class I) for over one year following resection. Table 1 shows the demographic and non-invasive findings for all patients. The age distribution differed between patients (mean  = 33.8, std  = 14.7) and controls (mean  = 36.4, stdev  = 13.3; t(49)  = 19.6, p<.01).

**Table 1:** Summary of patient demographics and clinical profile.

Pt	Clinical MRI Interpretation	Location	Age	Sex	Onset (years)	Seizure frequency	Scalp EEG
1	FCD	B occipital	53	m	12	8/year	B multifocal
2	FCD	L frontal	55	f	37	1/month	None
3	FCD	L parietal	12	m	7	4–8/day	B multifocal
4	FCD	R frontal	38	f	4	3/day	B multifocal
5	FCD	L temporal	46	f	5	1/month	L temporal
6	HT	R occipital	32	m	13	2/week	R temporo-central
7	EM	R temporal	39	m	20	1/month	R temporal
8	EM	L fronto-parietal	19	f	10	3/month	L central-parietal
9	EM	R occipital	40	f	7	1 cluster/month	R temporal
10	EM	R temporal	19	m	11	1 cluster/month	L fronto-central
11	PMG	B peri-sylvian	19	m	7	1/two weeks	L fronto-temporal-parietal

**Clinical MRI:** diagnosis based on visual inspection of routine clinical MRI (FCD: focal cortical dysplasia; EM: encephalomalacia; PMG: polymicrogyria; HT: heterotopia); **Location:** (Lobe, R =  right hemisphere, L =  left hemisphere, B =  bilateral); **Scalp EEG:** seizure onset location based on scalp video-EEG monitoring.

### Effect of smoothing

**Figure 3:**
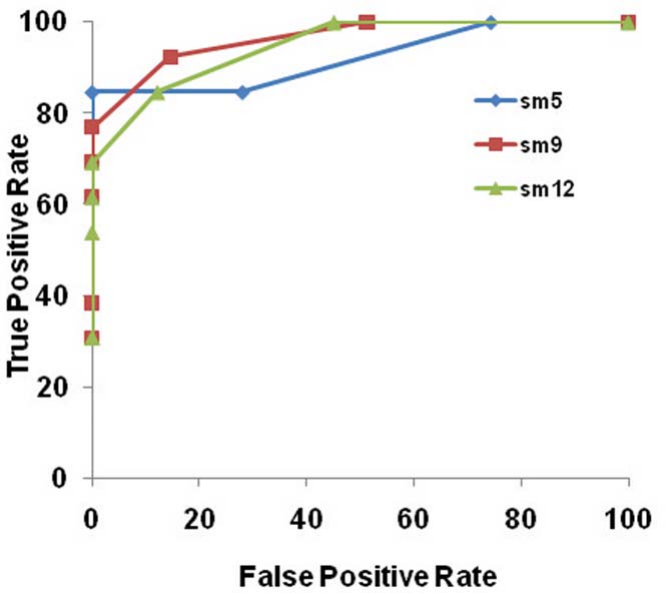
Receiver Operator Characteristics (ROC) curve showing effects of smoothing (smoothing level [sm] in FWHM [mm]) on sensitivity and specificity for thickness at different smoothing levels. Larger area under the curve shows higher discriminability.

Compared to threshold, smoothing affected the sensitivity and specificity, as well as the detection rate of the technique only marginally. ROC analysis showed better overall performance for sm  = 9 mm FWHM except in a very conservative environment where no false positives can be tolerated, where sm  = 5 mm FWHM performed best (Figure 3). However, these effects did not reach significance for overall detection sensitivity (ANOVA (*F*,(2, 28)  = 0.65, *p* = .52)) and specificity (*F*,(2, 28)  = 1.32, *p* = .27). Hence, all subsequent results presented here is for data smoothed at the lowest smoothing level.

### Effects of threshold

**Figure 4:**
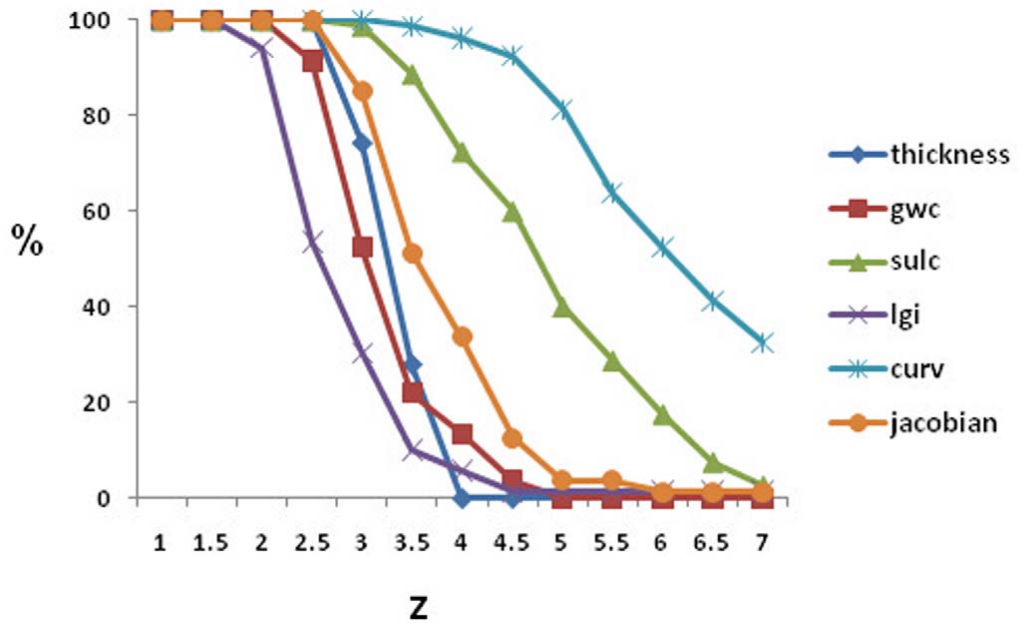
False positive clusters in normal control samples at different z-scores and different quantitative measures (gwc  =  grey-white contrast; sulc  =  sulcal depth; lgi  =  local gyrification index; curv  =  curvature; jacobia  =  jacobian distance). Calculations were performed for all controls (n = 41, 82 hemispheres).

Threshold had, predictably, a large effect on sensitivity (decreasing with increasing threshold) and specificity (increasing with increased threshold). Normal controls did not show any false positive clusters at z = 4 and above for cortical thickness, at z = 5 and above for GWC, at z = 4.5 for LGI, at z = 6 for jacobian distance and at z = 7 for sulcal depth. Curvature still showed false positives at z = 7 (Figure 4).

### Optimized Detection

**Figure 5:**
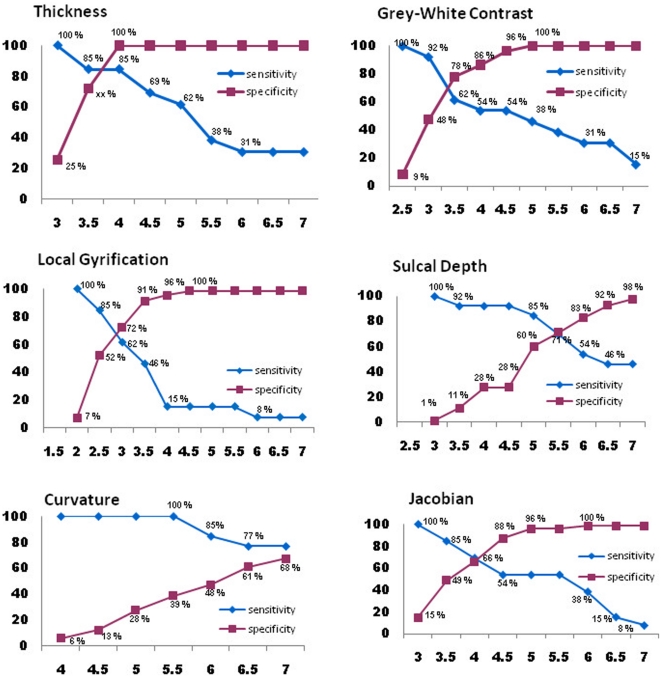
Detection sensitivity and specificity calculations for cortical surface measures calculated across patients and normal controls for different statistical thresholds (at FWHM  = 5 mm).

**Figure 6:**
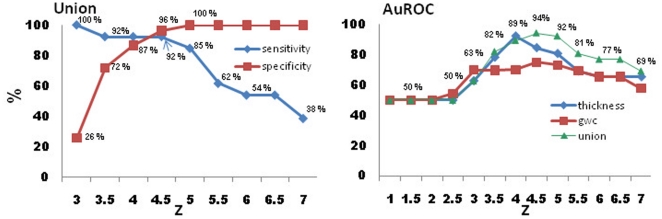
Sensitivity and specificity measures for the union (logical OR) of cortical thickness and grey-white contrast, and area-under-the-receiver-operator-curve (AuROC) calculations showing discriminability between patients and normal controls. Union measure combining thickness and contrast show superior performance.

Best performance trade-offs for sensitivity and specificity were found for cortical thickness and grey-white contrast (Figure 5), followed by, in descending order, LGI, sulcal depth and jacobian. Curvature showed the poorest performance. The detection threshold at which no control subject exhibited significant clusters (Figure 4) and that had the highest discriminability (Figure 5) was z  =  4 for cortical thickness and z = 3.5 for GWC. Sensitivity and specificity at these thresholds was, respectively, 100% and 84% for thickness, 61% and 78% for GWC. The best detection rate (96%) and sensitivity/specificity trade-off was achieved by combining both measures (union) (Figure 6), for which the optimal range for z-scores inlcude 4, 4.5 and 5, with a sensitivity/specificity of 92%/87% and 92%/96% and 85%/100%, respectively. Highest discriminatibility, as determined by area under the ROC curve, between patients and controls was 92% (*p*<.0001) for thickness (z = 4), 69% for GWC (*p*<.0001; z = 4.5), and 94% for Union (*p*>.0001; z = 4.5).

### Lesion coverage

**Figure 7:**
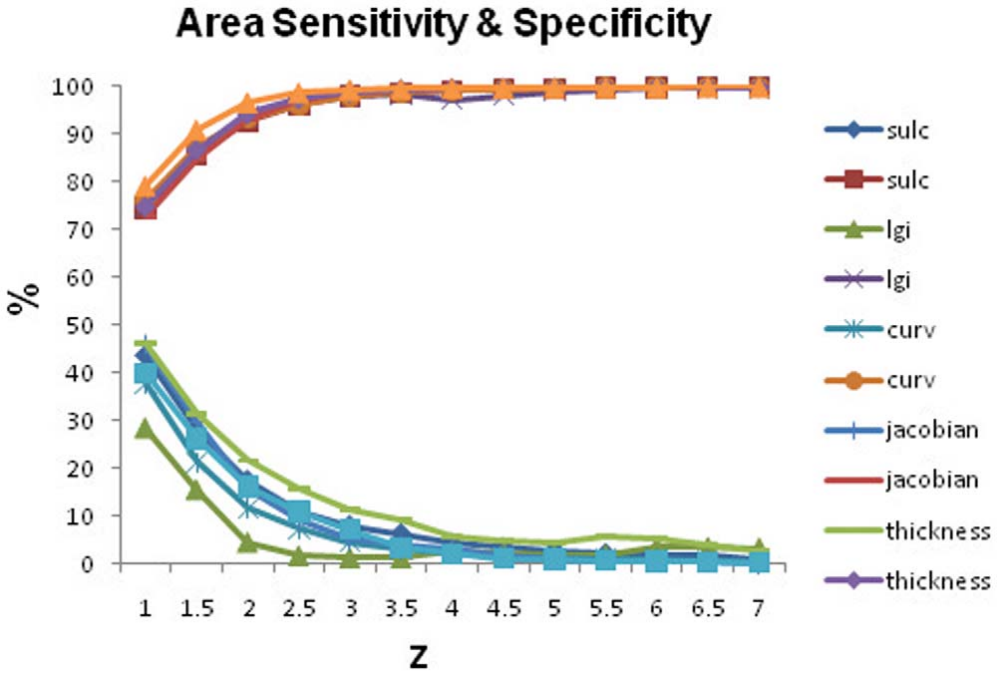
Sensitivity and specificity for quantitatively derived areas of abnormality compared to expert-delineated lesion margins for different thresholds.

Results from the lesion area-based sensitivity and specificity calculations are presented in Figure 7. The lesion coverage ratio is represented here by sensitivity.

### Correlation with Intracranial EEG and Pathology

Six of the patients were implanted with intracranial electrodes and subsequently underwent resective surgery of their epileptogenic zone as was clinically defined by regions of ictal and interictal activity as well as traditional neuroimaging results. The results of intracranial monitoring and surgery, along with the quantitative MRI detection findings, are summarized in Table 2. All patients were seizure free following resection with a median duration of follow-up of 1.7 years (0.5–2.6 years). All seizure onset zones, as derived from intracranial EEG monitoring, either overlapped or where immediately adjacent to the detected lesions in all surgical patients (classification criterion 1 [see Methods]). Post-operative pathology of the resected tissue also confirmed the presence of histological abnormalities in all of these patients. In addition to abnormal thickness and GWC measures within the lesion margin, 90% of patients (10/11) showed structural abnormalities outside the known lesion.

**Table 2:** Detected lesions for three different measurements (z>4) and combined extent (surface area in mm^2^) of significantly abnormal clusters outside the known lesion (z>4) for each patient.

Pt	Cortical Thickness		Gray/White Matter Blurring		Union		iEEG co-localizes	Histology	Surgical Outcome
	Lesion Detected	Extra-lesional detections	Lesion Detected	Extra-lesional detections	Lesion Detected	Extra-lesional detections			Engel Class
1 (LH)	no	942	yes	312	yes	1255	yes - L occipital	dysplasia, heterotopia	1
1 (RH)	yes	0	yes	0	yes	0	n/a	n/a	
2	yes	106	no	0	yes	106	no surgery	no surgery	
3	yes	507	no	146	yes	507	no surgery	no surgery	
4	yes	113	no	0	yes	113	yes - R frontal	dysplasia, heterotopia	1
5	no	0	no	0	no	0	yes - L temporal	dysplasia	1
6	yes	408	yes	184	yes	592	no surgery	no surgery	
7	yes	0	yes	45	yes	45	yes - R temporal	white matter rarefaction & gliosis	1
8	yes	905	yes	262	yes	1161	yes - L frontal	dysplasia, heterotopia	1
9	yes	0	yes	77	yes	77	yes - R temporal	neoplasm	1
10	yes	392	yes	1053	yes	1230	no surgery	no surgery	
11 (LH)	yes	105	no	0	yes	105	no surgery	no surgery	
11 (RH)	yes	486	no	0	yes	486	no surgery	no surgery	

Seizure onset localizations based on intracranial EEG and their co-localization with the lesion zone, post-operative pathology from the seizure onset zone and epilepsy surgery outcome.

## Discussion

Using surface-based MRI methods and quantitative comparisons based on 6 morphological features in individual patients and a large normal control group, we found that cortical thickness and grey-white matter contrast were the best detectors for epileptogenic malformations in the present sample. Based on these two measurements, we were able to automatically detect cortical lesions in 92% of patients with varying cortical malformations, while at the same time maintaining a low probability of false positives (96% specificity). Discriminability between patients and healthy controls was equally high at 94%. We were further able to relate the detected lesions to seizure onset zones, abnormal cell types on histology and positive outcome in all patients who underwent surgery. By systematically investigating the effects of threshold on ROC characteristics, we have identified an optimal detection range for threshold (z = 4 to 4.5) when using thickness and contrast, or the combination of both.

By using morphological measures important to the visual, clinical detection of CM (i.e., cortical thickness and GWC) and systematically varying smoothing and threshold, the current study demonstrates the optimal combination of processing parameters that allows the best discriminatory power between normal controls and individual patients. Thickness measures contributed more to detection than GWC, but their combination provided the best detection rates. Local gyrification exhibited the best sensitivity-specificity trade-off at z = 3, with 72% and 62% respectively. Sulcal depth showed a sensitivity and specificity of 71% at z = 5.5, which was slightly better than jacobian distance at 66%. Curvature was the worst detector for cortical abnormalities.

The inclusion of several pathologies in the patient sample suggests that the method is applicable for detecting the most common cortical malformations present in the epilepsy population. However, systematic studies with larger samples of varying types of pathologies, and their correlation with specific MRI features, are needed to allow the classification of malformations based on quantitative MRI features.

This ‘proof-of-concept’ study demonstrates that surface-based MRI morphometry can detect known epileptogenic structural abnormalities in cortex with high sensitivity and specificity, supporting its potential use as a tool in diagnosis and planning of epilepsy surgery. However, more work on larger patient samples needs to be done in order to determine the effectiveness of surface-based quantitative methods and the added value it offers compared to or in conjunction with traditional MRI review and volume-based quantitative methods (i.e. VBM).

We present sensitivity and specificity metrics to allow the determination of the best combination of parameters for a given purpose. Since the optimal parameters for detection depend on a trade-off between false negative and false positive likelihood, the optimal choice varies between applications. For example, when planning the cortical placement of intracranial electrodes [22], a higher false negative rate can be tolerated because the cost is low (i.e. placement of extra electrodes over normal brain regions) and is associated with a higher rate of detecting true positives, thus favoring high sensitivity. To inform about the location of resection areas, on the other hand, the potential costs are high and a low false positive rate is of crucial importance, necessitating the selection of parameters guaranteeing high specificity. These quantitative techniques are proposed as an adjunct to the pre-surgical evaluation of patients with medication-resistant focal epilepsy. They allow for further confirmation of a “suspicious” area on visual analysis, thereby helping to identify regions which may benefit from closer visual scrutiny, or further scanning at higher field strength, to confirm subtle lesions [23]. Ultimately, this would allow for improved strategic placement of intracranial electrodes for electrophysiological confirmation.

The low coverage ratios for area-based detection results indicate that the surface-based method is not suited for describing the extent of the lesional area or epileptogenic region, and thus guide the resection boundaries. This is especially true since the epileptogenic tissue can extend beyond the obvious margins of structurally abnormal tissue. Other image processing approaches may be better suited for delineating lesion extent [24]. Since the present study did not correct for the known age-related changes in cortical thickness [25] and gray-white matter blurring [26], and used a control sample with a wider age distribution, age-corrections may have yielded more sensitive performance measures. A further limitation of our analysis approach is the choice of expert-delineated lesion margins, which does not take into account the potential existence of additional lesions that are not visually detectable. Although this represents the “gold standard” of lesion detection in clinical practice (i.e. visual analysis), it cannot account for MRI-occult lesions (i.e. lesions which are not visually seen on MRI). In fact, the majority of our patients showed multiple areas of abnormal thickening and/or blurring that did not correspond to visually-identifiable MRI abnormalities. It is possible that they either represent statistical or processing artifacts, or are indeed ‘unconfirmed positives’ that are detecting true structural abnormalities which are too subtle to be detected by conventional visual MRI methods [27]. Some of the “false positive” areas seen in this study were distant from the known lesion(s) (i.e. in a different lobe or even hemisphere) and may indeed represent true areas of epilepsy-related malformations, which has been reported previously in patients with CM [28]. But these regions were not involved in the ictal onset zone and were thus not resected. The seizure free outcomes in all surgical patients, therefore, suggest that these regions were not epileptogenic. Further prospective investigations are needed to determine the relation of these regions to structural pathology, cortical hyperexcitability, and ultimately seizures. Future studies should also investigate the relationship with MCD types as identified by pathology and quantitative imaging findings.

Since the absence of any detected lesion leads to poorer surgical outcome [6], accurate identification of a cortical lesion during the pre-operative evaluation for epilepsy surgery is of high clinical importance. Although high-resolution MRI has made it possible to detect MCDs non-invasively in epilepsy patients, current studies suggest that up to about 70-80% of FCD's still go undetected by visual inspection. A potential future benefit of automated, quantitative, surface-based analysis methods may be the localization of subtle lesions that are not detectable by visual analysis alone. This, however, remains to be tested and further investigations are needed that aim to detect occult lesions, i.e. those who have focality suspected by clinical features and EEG, but who show MRI-negative findings, and that are subsequently confirmed by post-operative pathology. If successful, these techniques can benefit patients with focal partial epilepsy and MRI-negative findings by improving identification of patients that may benefit from surgery, and by offering better targets for in-depth visual study and guidance for invasive electrode placement in epilepsy surgery.

## Methods

### Ethics Statement

The study was approved by the Institutional Review Board of New York University Langone Medical Center and adheres to the Declaration of Helsinki (1964, 2008). All subjects gave written consent before participating in the study.

### Subjects

We studied 11 patients (5 females, age range: 12–37) at the Comprehensive Epilepsy Center of New York University who presented with focal epilepsy and cortical structural abnormalities on clinical MRI (see Table 1 for patients' clinical characteristics). Control subjects consisted of 41 right-handed, neurologically healthy individuals (age range: 19 to 66 years; mean  = 36.4, stdev  = 13.3, 23 females).

### MRI scanning and image preprocessing

Imaging was performed at the New York University Center for Brain Imaging on a Siemens Allegra 3T scanner. All patients were scanned with a specialized T1 MRI sequence subsequent to their clinical scan. Image acquisitions included a conventional 3-plane localizer and a T1-weighted volume pulse sequence (TE  = 3.25 ms, TR  = 2530 ms, TI  = 1100 ms, flip angle  = 7 deg field of view (FOV)  = 256 mm, matrix  = 256×256, voxel size  = 1×1×1.3 mm, scan time: 8∶07 min). Acquisition parameters were optimized for increased gray/white matter image contrast. The imaging protocol was identical for all subjects studied. Two T1-weighted images, acquired from each participant, were rigid body registered to each other, averaged (to increase SNR) and reoriented into a common space, roughly similar to alignment based on the AC-PC line. Images were corrected for nonlinear warping caused by no-uniform fields created by the gradient coils. Image intensities were further normalized and made uniform with the FreeSurfer software package.

### Surface reconstruction

In order to quantify the morphological characteristics of the human cerebral cortex, the volumetric MRI scans were used to construct models of each subject's cortical surface using an automated procedure that involves (1) segmentation of the white matter, (2) tessellation of the gray/white matter boundary, (3) inflation of the folded surface tessellation, and (4) automatic correction of topological defects. These steps are described in detail elsewhere [16]. From the reconstructed surfaces, measures of cortical thickness were obtained using the procedure described by [17]. First, an estimate of the gray/white matter boundary was constructed by classifying all white matter voxels in the MRI volume. Then, the white matter surface was refined in order to obtain submillimeter accuracy in delineating the gray/white matter junction. The surface was then deformed outward to locate the pial surface. Sulcal and gyral features across individual subjects and each patient were aligned by morphing each brain to an average spherical representation that allows for accurate matching of cortical locations among participant, while minimizing metric distortion [17]. Each reconstruction was inspected visually and corrected manually, if necessary. The degree of manual intervention was only minimal and largely focused on areas near the ventricles, putamen and dura mater.

### Morphometric measures

Surface-based measurements were computed at each vertex for a normative healthy control group (n = 41, 82 hemispheres), as well as for each patient (n = 11, 13 lesional hemispheres). Estimates of cortical thickness were made by measuring (1) the shortest distance from each point on the white matter surface to the pial surface, and (2) the shortest distance from each point on the pial surface to the white matter surface. Cortical thickness at each vertex was computed as the average of the two values [17]. Gray/white matter contrast (GWC) was estimated by calculating the non-normalized T1 image intensity contrast ([gray – white]/[gray + white]) at 0.5mm above vs. below the gray/white interface with trilinear interpolation of the images. Values can range from -1 to zero, with values closer to zero indicating less contrast and thus more blurring of the gray/white boundary. Gyrification is the ratio of the amount of cortex buried within the sulcal folds to the area visible from the outer visible cortex (i.e. on the gyral crowns). Larger gyrification indicates increased folding, and this measure has been used extensively in studies of 2D coronal ex-vivo brain slices [29]. Here, we computed a local gyrification index (LGI) that quantifies gyrification in circular three-dimensional regions at each vertex across the entire pial surface [30]. Sulcal depth measures were based on routines that calculate the dot product of the movement vectors with the surface normal [31], yielding measures of the depth/height of each point above the average surface, a measure that has been used to study morphological abnormalities in other developmental disorders [32]. In addition, mean curvature was estimated at each vertex using standard Freesurfer routines, giving a measure of the ‘sharpness’ of cortical folding of a gyrus or within a sulcus. Finally, the jacobian distance is a measure of the spherical non-linear transform needed to warp a subject into register with the spherical average brain. As such, the measure is conceptually similar to deformation-based morphometry, as described by Ashburner et al. [33], with the difference that alignment occurs in spherical surface space and follows individual gyral and sulcal patterns [31]. Morphometric measures were mapped to the inflated surface of each brain reconstruction, allowing the optimal visualization in both sulcal and gyral regions across the entire neocortex without being obscured by cortical folding. Data were then smoothed on the tessellated surface with a Gaussian smoothing kernel. The kernel size was varied to explore the effects of smoothing on lesion detection.

To detect regions with abnormal morphology, statistical comparisons using the z-statistic and cluster-based thresholding to control for multiple comparisons and spurious false positives were performed between patients and normal controls on a vertex-by-vertex basis [33]. Detection of statistically significant clusters can be altered by the degree of smoothing utilized during the image processing as well as the threshold standard score (z-score) which is considered to be abnormal. In order to find the best combination of measurements and processing parameters, we considered all 6 measures as well as the union of the most commonly used metrics (thickness and contrast combined by logical OR), 13 different threshold (z = 1–7) and 3 smoothing levels (FWHM (mm)  =  5, 9, 12) in our analysis.

### Lesion tracing

An expert rater board-certified in neurology and neurophysiology who was blinded to the intracranial EEG data reviewed the clinical MRI report and manually traced the outer regions of the visible lesions on the morphometric T1-weighted 3D volume scan based on the lesional areas identified in the initial report. When available, the visual detection was aided by T2 FLAIR images from the standard clinical epilepsy MRI protocol. The manual tracings in volume space were subsequently projected onto the cortical surface by assigning each voxel to the nearest surface vertex. Because the surface has sub-voxel resolution, a morphological closing operation was used to fill in any unlabeled vertices. The manual label on the pial surface was used to assess the degree of overlap with significant clusters.

### Diagnostic sensitivity and specificity compared to normal controls

Sensitivity and specificity estimates were calculated for categorical detection across patients and all normal controls, as well as a subset of 13 control hemispheres to match the number of patient hemispheres. To assess detections in the control population, all control subjects were subjected to a ‘leave-one-out’ procedure; each control subject was compared to the entire control group less the subject of interest. True positives were defined as patients with above-threshold clusters in the lesion area, false positives as healthy controls with any above-threshold clusters, and false negatives patients with no clusters within the lesion margin (Sensitivity: [% of positives correctly identified  =  true positives/(true positives + false negatives]; Specificity: [% of negatives correctly identified  =  true negatives/(true negatives + false positives)]). We also calculated the area under the receiver operator curve (ROC) to further evaluate the ability of the method to discriminate between patients and controls and to determine the discriminative ability at different thresholds [34].

### Sensitivity and specificity for area measures

To assess the influence of threshold and smoothing level on the ability of the detection measures to describe the extent of a lesion, we calculated a lesion coverage ratio, defined as the area in the lesion with super-threshold vertices relative to the total lesion area. Furthermore, we calculated the sensitivity and specificity of the surface area measures for describing the lesion margin. True positives were defined as above-threshold vertices within the lesion margin, true negatives as below-threshold vertices outside the lesion, false negatives as below-threshold vertices within the lesion, and false positives as above-threshold vertices outside the lesion margin. The total surface area represented by these vertices was used for all calculations. Area-based sensitivity in this case indicates accurate description of the lesion extent (i.e. the percentage of correctly classified vertices within the lesion margin). Specificity represents the likelihood of correctly classifying extra-lesional (i.e. as true negative) vertices.

### Intracranial EEG

Of the patients, 54% (6/11) underwent implantation of intracranial electrodes to define their seizure onset zone in preparation for resective surgery. Intracranial EEG from grid, strip and depth-electrodes was recorded utilizing BMSI 6000 video-EEG systems (Nicolet, Madison, Wisconsin) with up to 128 simultaneous channels. In order to determine whether the intracranial EEG (iEEG) seizure onset zones corresponded to the lesion margin, we first localized the intracranial electrodes by co-registering the volumetric pre-operative MRI to an MRI obtained with the implanted electrodes (Wang et al., in preparation). Using the known electrode grid and strip geometry together with error-minimization techniques, an accurate localization of the electrodes in relation to cortical gyral and sulcal structures was obtained and projected onto the reconstructed pial surface to assess the correspondence between the structural and electrophysiological epileptogenic markers. A rater, blinded to the results of the iEEG monitoring, categorized each electrode as either (1) in/on the lesion (defined as 50% or more of the 4 mm diameter electrode contained within the boundaries of the lesion), (2) adjacent to the lesion (defined as 10 mm or less from the lesion), or (3) outside the lesion. The classified electrodes were then compared to the iEEG results, allowing the ictal onset zones to be defined as either (1) in/on, (2) adjacent to, or (3) outside the detected lesion. All patients who underwent iEEG monitoring had subsequent resective surgery. Histo-pathological analyses were performed on the resected tissue as part of the patients' clinical care and were reviewed to correlate with imaging findings.

## References

[1] Lerner JT, Salamon N, Hauptman JS, Velasco TR, Hemb M (2009). Assessment and surgical outcomes for mild type I and severe type II cortical dysplasia: A critical review and the UCLA experience.. Epilepsia.

[2] Barkovich AJ, Kuzniecky RI (1996). Neuroimaging of focal malformations of cortical development.. J Clin Neurophysiol.

[3] Palmini A, Najm I, Avanzini G, Babb T, Guerrini R (2004). Terminology and classification of the cortical dysplasias.. Neurology.

[4] Barkovich AJ, Kuzniecky RI, Dobyns WB (2001). Radiologic classification of malformations of cortical development. Curr. Opin.. Neurol.

[5] Besson P, Andermann F, Dubeau F, Bernasconi A (2008). Small focal cortical dysplasia lesions are located at the bottom of a deep sulcus.. Brain.

[6] Sisodiya SM (2004). Surgery for focal cortical dysplasia.. Brain.

[7] Sisodiya SM (2000). Surgery for malformations of cortical development causing epilepsy.. Brain.

[8] Hakimi AS, Spanaki MV, Schuh LA, Smith BJ, Schultz L (2008). A survey of neurologists' views on epilepsy surgery and medically refractory epilepsy.. Epilepsy & Behavior.

[9] Ashburner J, Friston KJ (2000). Voxel-based morphometry–the methods.. Neuroimage.

[10] Bernasconi N, Duchesne S, Janke A, Lerch J, Collins DL (2004). Whole-brain voxel-based statistical analysis of gray matter and white matter in temporal lobe epilepsy.. NeuroImage.

[11] Mehta S, Grabowski TJ, Trivedi Y, Damasio H (2003). Evaluation of voxel-based morphometry for focal lesion detection in individuals.. NeuroImage.

[12] Wilke M, Kassubek J, Ziyeh S, Schulze-Bonhage A, Huppertz HJ (2003). Automated detection of gray matter malformations using optimized voxel-based morphometry: a systematic approach.. NeuroImage.

[13] Bonilha L, Montenegro MA, Rorden C, Castellano G, Guerreiro MM (2006). Voxel-based morphometry reveals excess gray matter concentration in patients with focal cortical dysplasia.. Epilepsia.

[14] Besson P, Bernasconi N, Colliot O, Evans A, Bernasconi A (2008). Surface-based texture and morphological analysis detects subtle cortical dysplasia.. Med Image Comput Comput Assist Interv.

[15] Fischl B, Sereno MI, Tootell RB, Dale AM (1999). High-resolution intersubject averaging and a coordinate system for the cortical surface.. Hum Brain Mapp.

[16] Dale AM, Fischl B, Sereno MI (1999). Cortical surface-based analysis. I. Segmentation and surface reconstruction.. Neuroimage.

[17] Fischl B, Dale AM (2000). Measuring the thickness of the human cerebral cortex from magnetic resonance images.. Proceedings of the National Academy of Sciences of the United States of America.

[18] Kuperberg GR, Broome MR, McGuire PK, David AS, Eddy M (2003). Regionally localized thinning of the cerebral cortex in schizophrenia. Arch. Gen.. Psychiatry.

[19] Peterson BS, Warner V, Bansal R, Zhu H, Hao X (2009). Cortical thinning in persons at increased familial risk for major depression.. Proc Natl Acad Sci USA.

[20] Westlye LT, Walhovd KB, Dale AM, Espeseth T, Reinvang I (2009). Increased sensitivity to effects of normal aging and Alzheimer's disease on cortical thickness by adjustment for local variability in gray/white contrast: a multi-sample MRI study.. Neuroimage.

[21] McDonald CR, Hagler DJ, Ahmadi ME, Tecoma E, Iragui V (2008). Regional neocortical thinning in mesial temporal lobe epilepsy.. Epilepsia.

[22] Wellmer J, Parpaley Y, von Lehe M, Huppertz H (2010). Integrating magnetic resonance imaging postprocessing results into neuronavigation for electrode implantation and resection of subtle focal cortical dysplasia in previously cryptogenic epilepsy.. Neurosurgery.

[23] Madan N, Grant PE (2009). New directions in clinical imaging of cortical dysplasias.. Epilepsia.

[24] Bonilha L, Montenegro MA, Rorden C, Castellano G, Guerreiro MM (2006). Voxel-based morphometry reveals excess gray matter concentration in patients with focal cortical dysplasia.. Epilepsia.

[25] Fjell AM, Westlye LT, Amlien I, Espeseth T, Reinvang I (2009). High Consistency of Regional Cortical Thinning in Aging across Multiple Samples. Cereb.. Cortex.

[26] Salat D, Lee S, van der Kouwe A, Greve D, Fischl B (2009). Age-associated alterations in cortical gray and white matter signal intensity and gray to white matter contrast.. NeuroImage.

[27] Colliot O, Bernasconi N, Khalili N, Antel S, Naessens V (2006). Individual voxel-based analysis of gray matter in focal cortical dysplasia.. NeuroImage.

[28] Fauser S, Sisodiya SM, Martinian L, Thom M, Gumbinger C (2009). Multi-focal occurrence of cortical dysplasia in epilepsy patients.. Brain.

[29] Zilles K, Armstrong E, Schleicher A, Kretschmann H (1988). The human pattern of gyrification in the cerebral cortex.. Anatomy and Embryology.

[30] Schaer M, Cuadra M, Tamarit L, Lazeyras F, Eliez S (2008). A Surface-Based Approach to Quantify Local Cortical Gyrification.. Medical Imaging, IEEE Transactions on.

[31] Fischl B, Sereno MI, Dale AM (1999). Cortical surface-based analysis. II: Inflation, flattening, and a surface-based coordinate system.. Neuroimage.

[32] Kippenhan JS, Olsen RK, Mervis CB, Morris CA, Kohn P (2005). Genetic Contributions to Human Gyrification: Sulcal Morphometry in Williams Syndrome.. J Neurosci..

[33] Ashburner J, Hutton C, Frackowiak R, Johnsrude I, Price C (1998). Identifying global anatomical differences: Deformation-based morphometry.. Human Brain Mapping.

[34] Metz CE (1978). Basic principles of ROC analysis.. Semin Nucl Med.

